# 8-Chloro-4-cyclo­hexyl-2*H*-1,4-benzoxazin-3(4*H*)-one

**DOI:** 10.1107/S1600536809008423

**Published:** 2009-03-14

**Authors:** Zhu-Bo Li, Xiao-Yan He, Wen-Liang Dong, Dan-Dan Liao

**Affiliations:** a College of Pharmaceutical Sciences, Southwest University, Chongqing 400715, People’s Republic of China; b School of Pharmaceutical Sciences, Shandong University of Traditional Chinese Medicine, Jinan 250355, People’s Republic of China

## Abstract

In the crystal structure of title compound, C_14_H_16_ClNO_2_, the cyclo­hexyl ring is in a chair conformation. The molecules are connected into centrosymmetric dimers *via* weak C—H⋯O hydrogen bonds.

## Related literature

For related structures, see: Li *et al.* (2008 ▶); Zuo *et al.* (2008 ▶).
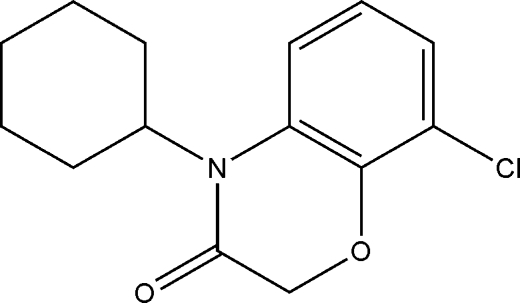


## Experimental

### 

#### Crystal data

C_14_H_16_ClNO_2_
*M* *_r_* = 265.73
Monoclinic, 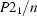

*a* = 9.0570 (8) Å
*b* = 5.7026 (5) Å
*c* = 25.289 (2) Å
β = 98.776 (1)°
*V* = 1290.8 (2) Å^3^
*Z* = 4
Mo *K*α radiation
μ = 0.29 mm^−1^
*T* = 293 K
0.12 × 0.10 × 0.06 mm

#### Data collection

Bruker SMART CCD area-detector diffractometer
Absorption correction: multi-scan (*SADABS*; Bruker, 2005 ▶) *T*_min_ = 0.967, *T*_max_ = 0.985
6491 measured reflections
2284 independent reflections
1865 reflections with *I* > 2σ(*I*)
*R*_int_ = 0.018

#### Refinement

*R*[*F* ^2^ > 2σ(*F* ^2^)] = 0.035
*wR*(*F* ^2^) = 0.094
*S* = 1.02
2284 reflections
163 parameters
H-atom parameters constrained
Δρ_max_ = 0.15 e Å^−3^
Δρ_min_ = −0.25 e Å^−3^

### 

Data collection: *SMART* (Bruker, 2005 ▶); cell refinement: *SAINT* (Bruker, 2005 ▶); data reduction: *SAINT*; program(s) used to solve structure: *SHELXS97* (Sheldrick, 2008 ▶); program(s) used to refine structure: *SHELXL97* (Sheldrick, 2008 ▶); molecular graphics: *XP* in *SHELXTL* (Sheldrick, 2008 ▶); software used to prepare material for publication: *SHELXL97*.

## Supplementary Material

Crystal structure: contains datablocks I, global. DOI: 10.1107/S1600536809008423/nc2137sup1.cif

Structure factors: contains datablocks I. DOI: 10.1107/S1600536809008423/nc2137Isup2.hkl

Additional supplementary materials:  crystallographic information; 3D view; checkCIF report

## Figures and Tables

**Table 1:** Hydrogen-bond geometry (Å, °)

*D*—H⋯*A*	*D*—H	H⋯*A*	*D*⋯*A*	*D*—H⋯*A*
C8—H8*A*⋯O2^i^	0.97	2.44	3.407 (3)	174

Symmetry code: (i) 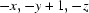.

**Fig. 1.**
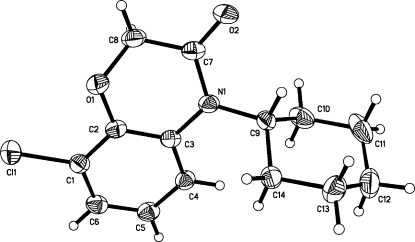
The molecular structure of the title compound with labeling and displacement ellipsoids drawn at the 50% probability level.

## supplementary crystallographic information

### Comment

As part of our project on the study of the interactions between small molecules and proteins (Li *et al.*; 2008 and Zuo *et al.*; 2008), we report here the synthesis and crystal structure of the title compound.

In the crystal structure of title compound, C_14_H_16_ClNO_2_, the cyclohexyl ring is in a chair conformation. The molecules are connected via two weak C-H···O hydrogen bonds into dimers which are located on centres of inversion.

### Experimental

To a solution of *N*-cyclohexyl-2-(2,3-dichlorophenoxy)acetamide(0.604 g, 2.0 mmol) in DMF (20 ml), caesium carbonate (0.787 g, 2.4 mmol) was added. The mixture was refluxed for 2 h. After completion of the reaction (by TLC monitoring), the DMF was removed under vacuum. Water (20 ml) was added into to obtain a turbid solution and it was extracted by ethyl acetate (20 ml *x* 4). The combined organic layer was washed by 1 mol/*L* of hydrochloric acid (10 ml *x* 3) and saturated sodium chloride solution (10 ml *x* 3), dried over MgSO~4~. And then the mixture was filtered and the filtrate obtained was concentrated under reduced pressure to obtain the corresponding crude product. The product was purified by column chromatography on silica gel using ethyl/acetate = 1/5 as eluent (yield 72%). Crystals suitable for X-ray diffraction were obtained by slow evaporation of a solutionof the solid dissolved in ethyl acetate/hexane at room temperature for 10 days.

### Refinement

All H atoms were palced in calculated positions and refined as riding, with C—H = 0.93–0.97Å and with *U*_iso_(H)=1.2Ueq(C).

### Crystal data

**Table:**

C_14_H_16_ClNO_2_	*F*(000) = 560
*M**_r_* = 265.73	*D*_x_ = 1.367 Mg m^−^^3^
Monoclinic, *P*2_1_/*n*	Mo *K*α radiation, λ = 0.71073 Å
Hall symbol: -P 2yn	Cell parameters from 2520 reflections
*a* = 9.0570 (8) Å	θ = 2.3–26.2°
*b* = 5.7026 (5) Å	µ = 0.29 mm^−^^1^
*c* = 25.289 (2) Å	*T* = 293 K
β = 98.776 (1)°	Block, colorless
*V* = 1290.8 (2) Å^3^	0.12 × 0.10 × 0.06 mm
*Z* = 4	

### Data collection

**Table:**

Bruker SMART CCD area-detector diffractometer	2284 independent reflections
Radiation source: fine-focus sealed tube	1865 reflections with *I* > 2σ(*I*)
graphite	*R*_int_ = 0.018
φ and ω scans	θ_max_ = 25.1°, θ_min_ = 2.3°
Absorption correction: multi-scan (*SADABS*; Bruker, 2005)	*h* = −10→10
*T*_min_ = 0.967, *T*_max_ = 0.985	*k* = −3→6
6491 measured reflections	*l* = −30→29

### Refinement

**Table:**

Refinement on *F*^2^	Primary atom site location: structure-invariant direct methods
Least-squares matrix: full	Secondary atom site location: difference Fourier map
*R*[*F*^2^ > 2σ(*F*^2^)] = 0.035	Hydrogen site location: inferred from neighbouring sites
*wR*(*F*^2^) = 0.094	H-atom parameters constrained
*S* = 1.02	*w* = 1/[σ^2^(*F*_o_^2^) + (0.0412*P*)^2^ + 0.4617*P*] where *P* = (*F*_o_^2^ + 2*F*_c_^2^)/3
2284 reflections	(Δ/σ)_max_ < 0.001
163 parameters	Δρ_max_ = 0.15 e Å^−^^3^
0 restraints	Δρ_min_ = −0.24 e Å^−^^3^

### Special details

**Table:**

Geometry. All e.s.d.'s (except the e.s.d. in the dihedral angle between two l.s. planes) are estimated using the full covariance matrix. The cell e.s.d.'s are taken into account individually in the estimation of e.s.d.'s in distances, angles and torsion angles; correlations between e.s.d.'s in cell parameters are only used when they are defined by crystal symmetry. An approximate (isotropic) treatment of cell e.s.d.'s is used for estimating e.s.d.'s involving l.s. planes.
Refinement. Refinement of *F*^2^ against ALL reflections. The weighted *R*-factor *wR* and goodness of fit *S* are based on *F*^2^, conventional *R*-factors *R* are based on *F*, with *F* set to zero for negative *F*^2^. The threshold expression of *F*^2^ > σ(*F*^2^) is used only for calculating *R*-factors(gt) *etc*. and is not relevant to the choice of reflections for refinement. *R*-factors based on *F*^2^ are statistically about twice as large as those based on *F*, and *R*- factors based on ALL data will be even larger.

### Fractional atomic coordinates and isotropic or equivalent isotropic displacement parameters (Å^2^)

**Table:**

	*x*	*y*	*z*	*U*_iso_*/*U*_eq_	
Cl1	−0.15312 (6)	0.41531 (10)	0.22217 (2)	0.06310 (19)	
O1	−0.02832 (15)	0.3875 (2)	0.12160 (5)	0.0532 (3)	
O2	0.13476 (15)	0.2157 (3)	0.01039 (5)	0.0598 (4)	
N1	0.18465 (15)	0.0827 (3)	0.09632 (5)	0.0423 (4)	
C1	−0.02699 (18)	0.2077 (3)	0.20691 (7)	0.0440 (4)	
C2	0.02332 (18)	0.2168 (3)	0.15761 (6)	0.0409 (4)	
C3	0.12797 (18)	0.0547 (3)	0.14560 (6)	0.0389 (4)	
C4	0.17380 (19)	−0.1228 (3)	0.18194 (7)	0.0447 (4)	
H4	0.2398	−0.2371	0.1736	0.054*	
C5	0.1216 (2)	−0.1303 (4)	0.23061 (7)	0.0500 (5)	
H5	0.1542	−0.2486	0.2550	0.060*	
C6	0.0224 (2)	0.0345 (4)	0.24338 (7)	0.0486 (5)	
H6	−0.0112	0.0294	0.2763	0.058*	
C7	0.0994 (2)	0.1974 (3)	0.05491 (7)	0.0459 (4)	
C8	−0.0428 (2)	0.3028 (4)	0.06762 (7)	0.0556 (5)	
H8A	−0.0722	0.4315	0.0432	0.067*	
H8B	−0.1213	0.1855	0.0621	0.067*	
C9	0.33367 (19)	−0.0012 (3)	0.08766 (7)	0.0408 (4)	
H9	0.3555	0.0833	0.0560	0.049*	
C10	0.3391 (3)	−0.2597 (4)	0.07334 (8)	0.0605 (6)	
H10A	0.2618	−0.2945	0.0434	0.073*	
H10B	0.3211	−0.3547	0.1035	0.073*	
C11	0.4918 (3)	−0.3181 (4)	0.05863 (9)	0.0836 (8)	
H11A	0.4973	−0.4852	0.0521	0.100*	
H11B	0.5037	−0.2370	0.0258	0.100*	
C12	0.6170 (3)	−0.2494 (5)	0.10186 (10)	0.0847 (8)	
H12A	0.7118	−0.2796	0.0898	0.102*	
H12B	0.6126	−0.3447	0.1333	0.102*	
C13	0.6084 (2)	0.0065 (5)	0.11650 (10)	0.0701 (6)	
H13A	0.6247	0.1024	0.0862	0.084*	
H13B	0.6868	0.0420	0.1460	0.084*	
C14	0.45753 (19)	0.0669 (3)	0.13235 (7)	0.0482 (5)	
H14A	0.4451	−0.0164	0.1648	0.058*	
H14B	0.4527	0.2337	0.1393	0.058*	

### Atomic displacement parameters (Å^2^)

**Table:**

	*U*^11^	*U*^22^	*U*^33^	*U*^12^	*U*^13^	*U*^23^
Cl1	0.0551 (3)	0.0751 (4)	0.0607 (3)	0.0114 (3)	0.0140 (2)	−0.0067 (3)
O1	0.0570 (8)	0.0561 (8)	0.0459 (7)	0.0134 (6)	0.0059 (6)	0.0113 (6)
O2	0.0662 (9)	0.0766 (10)	0.0368 (7)	0.0075 (7)	0.0084 (6)	0.0158 (7)
N1	0.0397 (8)	0.0521 (9)	0.0352 (7)	0.0022 (7)	0.0059 (6)	0.0101 (6)
C1	0.0338 (9)	0.0557 (11)	0.0421 (9)	−0.0043 (8)	0.0048 (7)	−0.0025 (8)
C2	0.0360 (9)	0.0455 (10)	0.0396 (9)	−0.0031 (8)	0.0008 (7)	0.0052 (8)
C3	0.0348 (8)	0.0466 (10)	0.0344 (8)	−0.0051 (7)	0.0026 (7)	0.0053 (7)
C4	0.0419 (9)	0.0481 (11)	0.0445 (9)	0.0028 (8)	0.0075 (8)	0.0084 (8)
C5	0.0482 (10)	0.0590 (12)	0.0426 (10)	−0.0023 (9)	0.0070 (8)	0.0165 (9)
C6	0.0434 (10)	0.0650 (13)	0.0385 (9)	−0.0073 (9)	0.0094 (8)	0.0047 (9)
C7	0.0465 (10)	0.0502 (11)	0.0392 (10)	−0.0027 (8)	0.0005 (8)	0.0090 (8)
C8	0.0501 (11)	0.0736 (14)	0.0413 (10)	0.0074 (10)	0.0009 (8)	0.0161 (10)
C9	0.0457 (10)	0.0435 (10)	0.0344 (8)	0.0037 (8)	0.0097 (7)	0.0025 (7)
C10	0.0896 (16)	0.0463 (12)	0.0438 (10)	0.0013 (11)	0.0047 (10)	−0.0079 (9)
C11	0.143 (2)	0.0577 (14)	0.0608 (14)	0.0345 (15)	0.0488 (16)	0.0026 (11)
C12	0.0815 (17)	0.102 (2)	0.0784 (16)	0.0455 (16)	0.0383 (14)	0.0203 (15)
C13	0.0440 (11)	0.0946 (18)	0.0733 (14)	0.0063 (12)	0.0146 (10)	0.0075 (13)
C14	0.0447 (10)	0.0530 (12)	0.0472 (10)	−0.0001 (9)	0.0082 (8)	−0.0058 (9)

### Geometric parameters (Å, °)

**Table:**

Cl1—C1	1.7292 (19)	C8—H8B	0.9700
O1—C2	1.366 (2)	C9—C14	1.516 (2)
O1—C8	1.435 (2)	C9—C10	1.521 (3)
O2—C7	1.221 (2)	C9—H9	0.9800
N1—C7	1.369 (2)	C10—C11	1.523 (3)
N1—C3	1.427 (2)	C10—H10A	0.9700
N1—C9	1.479 (2)	C10—H10B	0.9700
C1—C6	1.378 (3)	C11—C12	1.502 (4)
C1—C2	1.392 (2)	C11—H11A	0.9700
C2—C3	1.390 (2)	C11—H11B	0.9700
C3—C4	1.388 (2)	C12—C13	1.510 (4)
C4—C5	1.385 (2)	C12—H12A	0.9700
C4—H4	0.9300	C12—H12B	0.9700
C5—C6	1.372 (3)	C13—C14	1.521 (3)
C5—H5	0.9300	C13—H13A	0.9700
C6—H6	0.9300	C13—H13B	0.9700
C7—C8	1.500 (3)	C14—H14A	0.9700
C8—H8A	0.9700	C14—H14B	0.9700
			
C2—O1—C8	111.50 (15)	N1—C9—H9	105.3
C7—N1—C3	119.02 (14)	C14—C9—H9	105.3
C7—N1—C9	117.53 (14)	C10—C9—H9	105.3
C3—N1—C9	123.43 (13)	C9—C10—C11	109.46 (18)
C6—C1—C2	120.58 (17)	C9—C10—H10A	109.8
C6—C1—Cl1	119.96 (14)	C11—C10—H10A	109.8
C2—C1—Cl1	119.46 (14)	C9—C10—H10B	109.8
O1—C2—C3	120.24 (15)	C11—C10—H10B	109.8
O1—C2—C1	119.88 (16)	H10A—C10—H10B	108.2
C3—C2—C1	119.87 (16)	C12—C11—C10	112.22 (17)
C4—C3—C2	119.04 (15)	C12—C11—H11A	109.2
C4—C3—N1	123.27 (16)	C10—C11—H11A	109.2
C2—C3—N1	117.69 (14)	C12—C11—H11B	109.2
C5—C4—C3	120.20 (17)	C10—C11—H11B	109.2
C5—C4—H4	119.9	H11A—C11—H11B	107.9
C3—C4—H4	119.9	C11—C12—C13	111.6 (2)
C6—C5—C4	120.82 (17)	C11—C12—H12A	109.3
C6—C5—H5	119.6	C13—C12—H12A	109.3
C4—C5—H5	119.6	C11—C12—H12B	109.3
C5—C6—C1	119.38 (16)	C13—C12—H12B	109.3
C5—C6—H6	120.3	H12A—C12—H12B	108.0
C1—C6—H6	120.3	C12—C13—C14	111.4 (2)
O2—C7—N1	123.31 (17)	C12—C13—H13A	109.3
O2—C7—C8	121.21 (16)	C14—C13—H13A	109.3
N1—C7—C8	115.47 (15)	C12—C13—H13B	109.3
O1—C8—C7	112.45 (14)	C14—C13—H13B	109.3
O1—C8—H8A	109.1	H13A—C13—H13B	108.0
C7—C8—H8A	109.1	C9—C14—C13	109.76 (16)
O1—C8—H8B	109.1	C9—C14—H14A	109.7
C7—C8—H8B	109.1	C13—C14—H14A	109.7
H8A—C8—H8B	107.8	C9—C14—H14B	109.7
N1—C9—C14	113.26 (14)	C13—C14—H14B	109.7
N1—C9—C10	114.32 (16)	H14A—C14—H14B	108.2
C14—C9—C10	112.25 (15)		
			
C8—O1—C2—C3	36.1 (2)	C3—N1—C7—O2	−175.04 (17)
C8—O1—C2—C1	−145.10 (16)	C9—N1—C7—O2	6.4 (3)
C6—C1—C2—O1	179.06 (16)	C3—N1—C7—C8	5.2 (2)
Cl1—C1—C2—O1	−0.7 (2)	C9—N1—C7—C8	−173.32 (16)
C6—C1—C2—C3	−2.1 (3)	C2—O1—C8—C7	−54.4 (2)
Cl1—C1—C2—C3	178.15 (13)	O2—C7—C8—O1	−145.51 (18)
O1—C2—C3—C4	−177.29 (15)	N1—C7—C8—O1	34.2 (2)
C1—C2—C3—C4	3.9 (2)	C7—N1—C9—C14	130.77 (17)
O1—C2—C3—N1	3.6 (2)	C3—N1—C9—C14	−47.7 (2)
C1—C2—C3—N1	−175.20 (15)	C7—N1—C9—C10	−98.95 (19)
C7—N1—C3—C4	155.62 (17)	C3—N1—C9—C10	82.6 (2)
C9—N1—C3—C4	−25.9 (3)	N1—C9—C10—C11	172.94 (15)
C7—N1—C3—C2	−25.3 (2)	C14—C9—C10—C11	−56.3 (2)
C9—N1—C3—C2	153.11 (16)	C9—C10—C11—C12	54.7 (2)
C2—C3—C4—C5	−3.4 (3)	C10—C11—C12—C13	−55.0 (3)
N1—C3—C4—C5	175.67 (16)	C11—C12—C13—C14	55.3 (3)
C3—C4—C5—C6	1.0 (3)	N1—C9—C14—C13	−171.56 (16)
C4—C5—C6—C1	0.8 (3)	C10—C9—C14—C13	57.1 (2)
C2—C1—C6—C5	−0.3 (3)	C12—C13—C14—C9	−55.7 (2)
Cl1—C1—C6—C5	179.49 (14)		

### Hydrogen-bond geometry (Å, °)

**Table:**

*D*—H···*A*	*D*—H	H···*A*	*D*···*A*	*D*—H···*A*
C8—H8A···O2^i^	0.97	2.44	3.407 (3)	174

Symmetry codes: (i) −*x*, −*y*+1, −*z*.
